# Adsorption Study of Lignin Removal from Recycled Alkali Black Liquor by Adsorption Resins for Improved Cellulase Hydrolysis of Corn Straw

**DOI:** 10.3390/molecules25194475

**Published:** 2020-09-29

**Authors:** Yiming Li, Shuo Fang, Xia Zhou, Zhezhen Zhao, Fei Li, Ping Liu

**Affiliations:** College of Food Science and Nutritional Engineering, China Agricultural University, Beijing 100083, China; liyimingbest@163.com (Y.L.); shuofangssdd@163.com (S.F.); 15008139362@163.com (X.Z.); zhaozz9789@163.com (Z.Z.); dayya516@163.com (F.L.)

**Keywords:** adsorption resin, lignin, recycled alkali black liquor, adsorption, cellulase hydrolysis

## Abstract

Previous studies showed that aromatic compounds such as lignin, phenols, and furans were main inhibitors of cellulase hydrolysis in recycled alkali black liquor (RBL), which should be removed to improve alkali utilization. In this study, three polymeric resins, XAD-4, XAD-16N, and XAD-7HP, were evaluated for their abilities to remove lignin from alkali black liquor recycled at the third time. Adsorption conditions of adsorbent dose and equilibrium time, isotherms, and kinetics were investigated. Of three tested adsorbents, XAD-16N was the most efficient, which can remove 89.84% of lignin after adsorption at an adsorbent-to-solution ratio of 1:4 for 2.5 h. Pseudo-second-order model was efficient to represent XAD-16N and XAD-7HP adsorption kinetics. Adsorption behavior of XAD-4 on RBL was fitted better to Langmuir model, while XAD-16N and XAD-7HP adsorption were more consistent with Freundlich model. The cellulase hydrolysis rate of corn straw treated with RBL after XAD-16N adsorption combined with ozone was 86.89%, which was only 0.89% lower than that of sodium hydroxide combined with ozone treatment. Structure characterization proved that the damage of XAD-16N adsorbed RBL to corn straw was similar to that of sodium hydroxide. It indicated that adsorption was effective in inhibitor removal from RBL to improve alkali utilization.

## 1. Introduction

Alkali black liquor produced by corn straw treatment contains alkali, inorganic matters, and organic chemicals, such as polysaccharides, lignin, and cross-linked macromolecules composed of aromatic groups, which has significant impacts on the environment as the toxic and hazardous waste [1,2]. It is necessary to recycle alkali black liquor to protect environment, save resources, and improve economic benefit [3]. Cha et al. investigated the feasibility of recycled alkali black liquor (RBL) generated by twin-screw extrusion pretreatment and found that glucose yields from the Miscanthus biomass pretreated with the first and second circulated black liquor were 96.62% and 83.26% of that treated with NaOH. A total of 69.70% of NaOH was saved after the first black liquor treatment [4]. Goshadrou found that RBL treatment of Cogongrass for ten times could save 45% of the alkali consumption and 59% of the water usage, and the final enzymatic hydrolysis yields of biomass were in the range of 66.40–88.70%, which showed a downward trend by further recycling [1]. Previous laboratory studies have found that the lignin removal and cellulase hydrolysis efficiency of the corn straw treated by RBL gradually decreased with the increasing cycle times, and the optimal circulation time was the third time [5]. The components of RBL were studied in the previous study, and it was found that aromatic compounds, such as lignin, phenols, benzene ring heterocycles and furans were the main inhibitors of cellulase hydrolysis, which accumulated with circulation. Lignin could impede enzymatic hydrolysis by limiting the accessibility of polysaccharides and non-productive binding to enzymes. As one of the significant recalcitrance contributors, the presence of lignin in RBL hindered the conversion of biomass and reduced the RBL treatment efficiency [6]. Therefore, RBL needs to purify to remove the inhibitors to improve utilization of alkali.

The main purification methods are biological, chemical, and physical methods. Commonly used microorganisms in biological methods are fungus, such as white rot fungi, which can secrete extracellular enzymes and possess good activity to degrade lignin and toxic phenolic compounds [7]. However, alkali black liquor is not conducive to fungal growth and enzyme production because of high pH and low dissolved oxygen [8]. The disadvantages of biological method are that they have low efficiency and are time-consuming [9]. Chemical methods mainly include precipitation by changing pH or temperature of the solution [10], organic solvent extraction [11], and electrochemical [12]. Physical methods include membrane filtration technology such as ultrafiltration and nanofiltration [13], evaporation concentration [14], adsorption and so on. Among these methods, adsorption is preferred due to low-cost, easy operation, environmental protection, energy-saving [15,16] and its high efficiency and selectivity to the purification of complex systems [17]. Various adsorbents, including activated carbon [18], ion exchange resins [19,20] and adsorption resins [9,10,17] have been extensively studied for removing lignin and other organics from lignocellulose hydrolysate. As a traditional adsorbent, the drawbacks of activated carbon are high production cost, difficult regeneration caused by strong adsorption capacity with adsorbed compounds, and the production of carbons fines due to the brittleness of carbons [10,20]. The regeneration of ion exchange resin demands a large number of acids and alkalis, which produces lots of wastewaters [21]. Adsorption resin is a novel type of polymeric adsorbent [9], which possesses chemical stability, better selectivity, and high adsorption capacity and can be regenerated by simple and non-destructive methods [20]. The adsorption resin is an efficient choice for the removal of aromatic impurities [10,17], which can adsorb phenols and furans, etc. from lignocellulose hydrolysate by Van der Waals force [22].

Amberlite XAD-4, XAD-16N, XAD-7HP resins are non-polar macroporous adsorption resin [23], which have been applied to remove lignin and its degradation from lignocellulose hydrolysate [24]. Heinonen et al. [10] used six commercial polymeric adsorbents to separate and recover lignin from monosaccharide rich hydrolysates of lignocellulose and found that the XAD-16N resin was the most efficient with 80% lignin removal. You et al. [25] studied the adsorption of lignin in the alkali-extracted hemicellulose from bagasse on XAD-16N resin and found out that the delignification rate reached 89.2%. Schwartz and Lawoko [26] reported that XAD-4 adsorption could remove 100% of furan derivatives and 90% of acid-soluble lignin from the extracts of mixed hardwood chips. Padilha et al. [27] discovered that 72.33% phenolic compounds could be removed from hydrothermal pretreatment liquor of green coconut fiber by using XAD-7HP resin. Koivula et al. [28] used XAD-16 and XAD-7HP resin to purify wood autohydrolysates as the pretreatment for ultrafiltration and found that both resins could effectively remove ligneous material, thereby enhancing ultrafiltration capability. The lignin removal rate of XAD-16 and XAD-7HP were 70% and 50%, respectively.

According to previous laboratory research [5], the appropriate cycle time of alkali black liquor was the third time. In this study, the above three kinds of resins were used to purify alkali black liquor recycled at the third time. Lignin was used as an indicator to measure the adsorption efficiency of polymeric resins, the adsorption conditions such as adsorbent dosage and adsorbent time were investigated systematically. The adsorption isotherms and kinetics were analyzed to understand the adsorption mechanism. Then the purified alkali black liquor was combined with ozone to treat the corn straw. The effect of alkali black liquor purification on the enzymatic hydrolysis of corn straw was studied by structural characterization and determination of cellulase hydrolysis rate. This study aims to provide the appropriate adsorbent resin and obtain its optimal adsorption conditions for the purification and utilization of RBL.

## 2. Results and Discussion

### 2.1. Effect of Adsorption Time on Lignin Removal from Alkali Black Liquor

**Figure 1:**
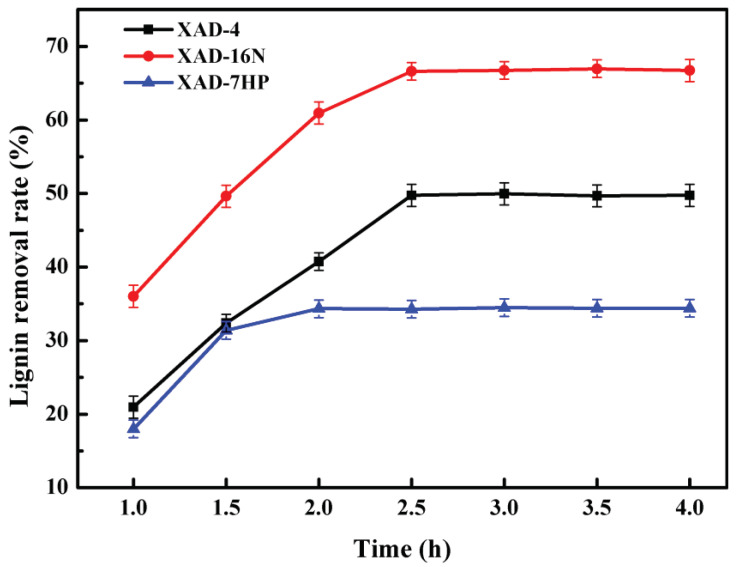
Effect of adsorption time on lignin removal from alkali black liquor.

The effect of adsorption time of three different polymer resins on lignin removal is shown in Figure 1. It could be seen that the lignin removal rate of three different adsorbents increased rapidly with the adsorption time at first, then the rate of lignin adsorption became slower, and no further high delignification rate was found after the equilibrium was reached. The delignification capacity of XAD-4, XAD-16N and XAD-7HP resins increased with increasing contact time up to 2.5 h, 2.5 h and 2 h, respectively, and then it was nearly constant. The adsorption capacity of XAD-16N was better than that of XAD-4 and XAD-7HP and the maximum delignification rate of XAD-16N was 66.62%, which was 16.88% higher than XAD-4 and 32.29% higher than XAD-7HP, respectively.

### 2.2. Effect of Adsorbent Dosage on Lignin Removal from Alkali Black Liquor

**Figure 2:**
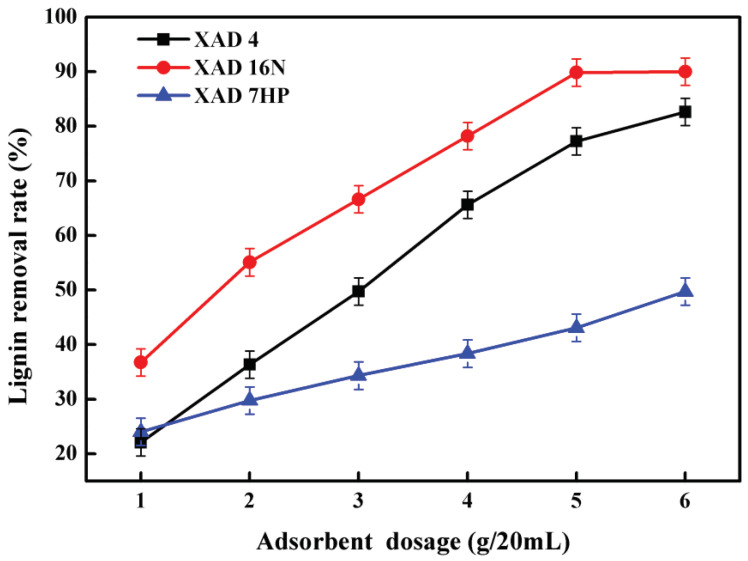
Effect of adsorbent dosage on lignin removal from alkali black liquor.

The effect of adsorbent dosage in the range of 1–6 g/20mL on lignin removal of three different adsorbents was investigated. As shown in Figure 2, the removal efficiency of lignin increased with an increase in the adsorbent dosage. Among these three kinds of resins, XAD-16N exhibited the best capacity of delignification, followed by XAD-4 and XAD-7HP. The rate of lignin removal after XAD-16N adsorption was 36.74% at the dosage of 1 g/20 mL, which increased rapidly and reached the maximum of 89.84% at 5 g/20 mL. The extent of lignin adsorption efficiency increased by increasing the XAD-4 and XAD-7HP resin amount. The delignification rate of XAD-4 was 22.10% at the dosage of 1 g/20 mL, which was 1.94% lower than XAD-7HP and increased to 82.63% at the dosage of 6 g/20 mL which was 32.90% higher than XAD-7HP.

It could be found that the lignin removal efficiency of XAD-16N, XAD-4 and XAD-7HP resins increased by increasing the resin amount, but the removal rate of XAD-16N and XAD-4 increased slowly. This phenomenon can be explained by the fact that the adsorption sites increased with rising adsorbent dosage and the lignin adsorption on the resin is quickly saturated at small resin amount, resulting in an increase of delignification rate [16,25]. However, the content of lignin that is easy to adsorb is decreased with the increasing resin dosage, and the remaining lignin in alkali black liquor is hard to adsorb, even if the resin adsorption has not reached saturation, which lead to a slower increase in delignification rate [25]. Among the three adsorbents, XAD-7HP showed the lowest lignin removal rate, and the optimal dosage was higher than 6 g/20 mL. The results were similar with You et al. [25] and Heinonen et al. [10], XAD-16N exhibited good adsorption capacity with the lignin removal rate of over 80%.

### 2.3. Adsorption Kinetics Studies for Lignin from Alkali Black Liquor

In order to investigate and explain the behavior of lignin in RBL on XAD-4, XAD-16N and XAD-7HP resins, the pseudo-first-order and pseudo-second-order kinetic models were fitted.

**Figure 3:**
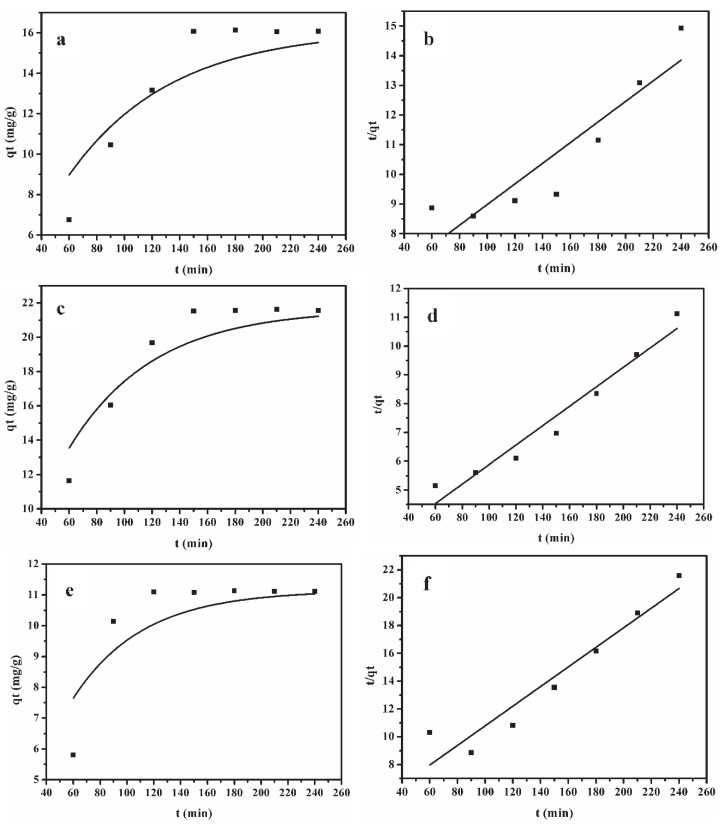
Pseudo-first-order (**a**,**c**,**e**) and pseudo-second-order (**b**,**d**,**f**) kinetic models of lignin adsorption on different resins. (**a**,**b**) XAD-4 resin adsorption; (**c**,**d**) XAD-16N resin adsorption; (**e**,**f**) XAD-7HP resin adsorption.

Figure 3 presented the fitting curves of pseudo-first-order (*q_t_* versus t) and pseudo-second-order models (t/*q_t_* versus t) of the three different adsorption resins. The values of *k*_1_, *k*_2_ and the corresponding linear regression correlation coefficient R^2^ were listed in Table 1. It can be observed that R^2^ of pseudo-second-order model was higher than that of pseudo-first-order model for XAD-16N and XAD-7HP resins, indicating that pseudo-second-order model was more suitable to describe the adsorption of lignin from recycled black liquor by XAD-16N and XAD-7HP polymeric resins. Pseudo-first-order model could better describe the behavior of XAD-4 resin adsorption. The second-order kinetics suggested that the adsorption of lignin by XAD-16N and XAD7-HP in RBL was a chemisorption process [29]. The pseudo-second-order model is based on the reaction caused by sharing or exchange of electrons [15,29], therefore, the rate-limiting step of the entire adsorption process was caused by electron sharing or exchange between the cross-linking group and the alkali black liquor.

### 2.4. Adsorption Mechanism

It is generally believed that the resin adsorption process can be divided into three steps, liquid film diffusion, intraparticle diffusion and adsorption process. In general, the resin adsorption can be controlled by either the liquid film diffusion or intraparticle diffusion [15]. In Weber&Morris diffusion model, it is assumed that lignin adsorption occurs in three process: (1) the migration of lignin from RBL to the surface of the adsorbent, (2) the diffusion of lignin from the outer surface to the inner pores of the adsorbent, (3) the attachment of lignin to the active sites on the pore surface of the particle by chemical reaction [30].

**Figure 4:**
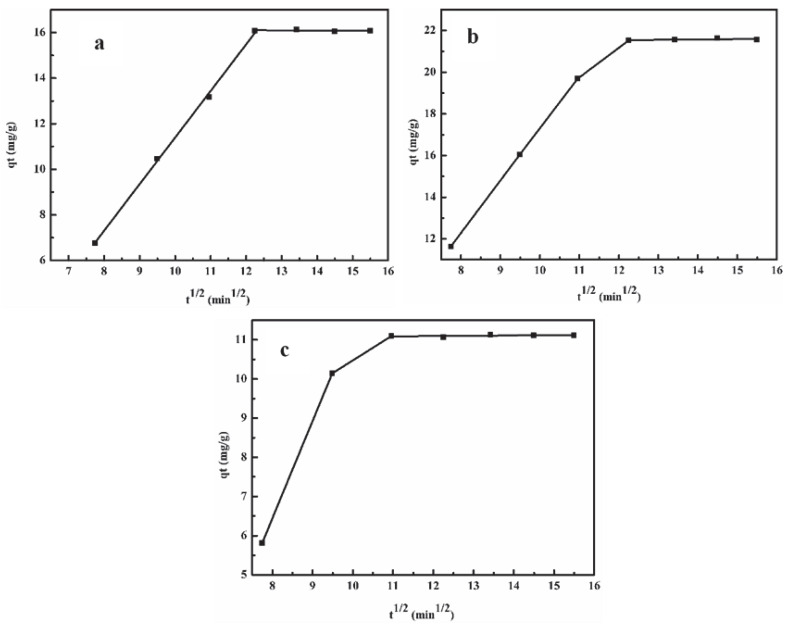
Intraparticle diffusion model. (**a**) XAD-4 resin adsorption; (**b**) XAD-16N resin adsorption; (**c**) XAD-7HP resin adsorption.

The linear fitting of *q_t_* versus t^1/2^ was shown in Figure 4. The linear-fitting exhibited multi-linear plots, indicating that lignin removal from RBL was controlled by different steps. The curves of XAD-16N and XAD-7HP adsorption could be divided into three stages, indicating that lignin removal from RBL was controlled by different steps. The first stage showed the external mass transfer with low resistance and high adsorption rate, lignin diffused to the resin surface. In the second stage, lignin migrated to adsorption sites, the outer surface adsorption saturated, and the interior surface adsorption increased, resulting in increased diffusion resistance and lower adsorption rate where the intraparticle diffusion was the rate-limiting step. The third stage was the lignin adsorption saturation tends to equilibrium. The fitting linear failed to pass the origin, which indicated that intraparticle diffusion was not the only rate-controlling step during the entire adsorption process [30].

### 2.5. Adsorption Isotherms for Lignin from Alkali Black Liquor

The adsorption isotherms are important to describe the interactive reactions between adsorbents and adsorbates [31]. Langmuir and Freundlich isotherms models were selected to analyze the equilibrium data of sorption of lignin on XAD-4, XAD-16N and XAD-7HP resins. The Langmuir isotherm is based on the assumption that a monolayer adsorption takes place on homogeneous surface containing a limited number of adsorption sites of uniform energy, and there is no interaction among the adsorbed molecules [32]. The Freundlich isotherm model is an empirical equation, which supposes that the multilayer sorption occurs on the heterogeneous surfaces and the interactions among the adsorbed molecules are considered [33]. 

**Figure 5:**
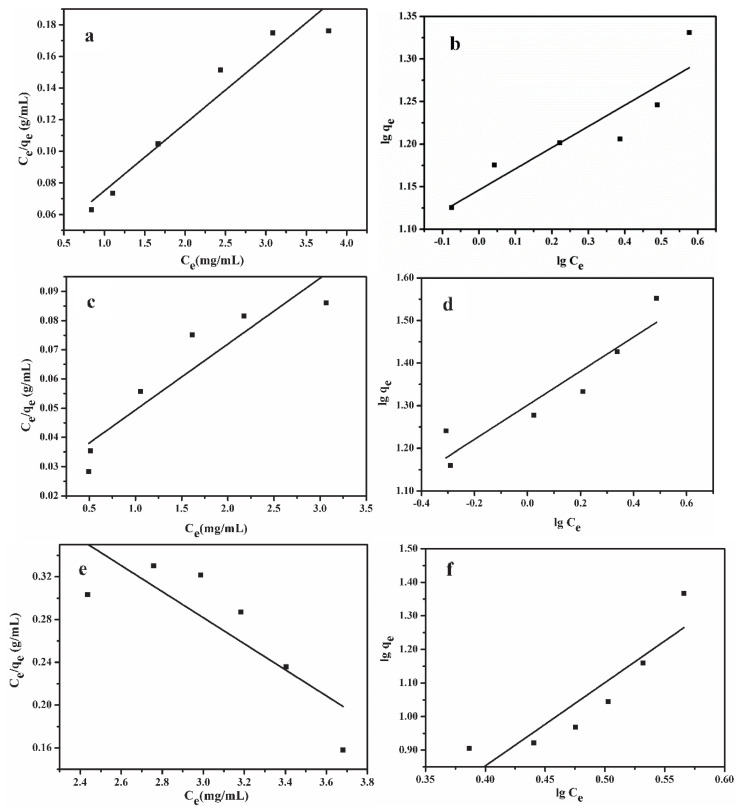
Langmuir (**a**,**c**,**e**) and Freundlich (**b**,**d**,**f**) adsorption isotherms for lignin on different resins. (**a**,**b**) XAD-4 resin adsorption; (**c**,**d**) XAD-16N resin adsorption; (**e**,**f**) XAD-7HP resin adsorption.

Figure 5 showed the curve fit to Langmuir isotherm models (*C_e_*/*q_e_* versus *C_e_*) and Freundlich isotherm models (lg *q_e_* versus lg *C_e_*) of three adsorption resins. The relevant parameters calculated from the two isotherm models were summarized in Table 2. As can be seen, the adsorption data of XAD-4 was well fitted to Langmuir isotherm model and Freundlich isotherm model was better suited to predict the adsorption by XAD-16N and XAD-7HP resin, by comparing the correlation coefficient (R^2^). It indicated that the adsorption of lignin on XAD-4 resin was a monolayer adsorption which distributed uniformly on the resin surface, and the adsorption of XAD-16N and XAD-7HP was multilayer adsorption. The Freundlich parameter 1/n for XAD-16N adsorption was less than 1, indicating favorable adsorption, while the value of 1/n for XAD-7HP adsorption was higher than 1, illustrating that the adsorption was unfavorable. The maximum saturated adsorption capacity of XAD-16N was higher than that of XAD-4.

It can also be observed that the experimental data of XAD-7HP adsorption are not well fitted to the two models, it might be that the adsorption equilibrium of XAD-7HP had not reached at the adsorption dose of 6 g/20 mL, which affected the data fitting. The XAD-7HP adsorption equilibrium conditions were not studied further in this study due to the smaller amount of XAD-4 and XAD-16N used.

### 2.6. Cellulase Hydrolysis of Treated Corn Straw

On the basis of the results presented above, XAD-16N was proved to be the most efficient and chosen for further studies. Preliminary laboratory experiments [5] found that the cellulase hydrolysis rate of corn straw treated with NaOH combined with ozone (NaOH-ozone) was (87.67 ± 0.88)%, and that of RBL at the fourth cycle combined with ozone (RBL4-ozone) treated corn straw was (76.27 ± 0.76)%. The cellulase hydrolysis rate of straw treated by XAD-16N adsorbed RBL at the fourth cycle combined with ozone (ARBL4-ozone) treatment was (86.89 ± 0.73)%, which increased by 13.92% compare with the unpurified RBL4-ozone treatment and was only 0.89% lower than fresh NaOH-ozone treatment. It can be concluded that XAD-16N can purify the RBL efficiently, large amount of lignin was adsorbed by XAD-16N resin, the effective alkali amount increased and the destructive ability of the alkali black liquor to lignocellulose was restored, resulting in improved cellulase hydrolysis rate. Meanwhile, compared with activated carbon and ion exchange resins, XAD-16N resin can be regenerated by ethanol at normal temperature and has high lignin removal capacity, which led to low cost and energy-saving.

### 2.7. SEM of Treated Corn Straw

**Figure 6:**
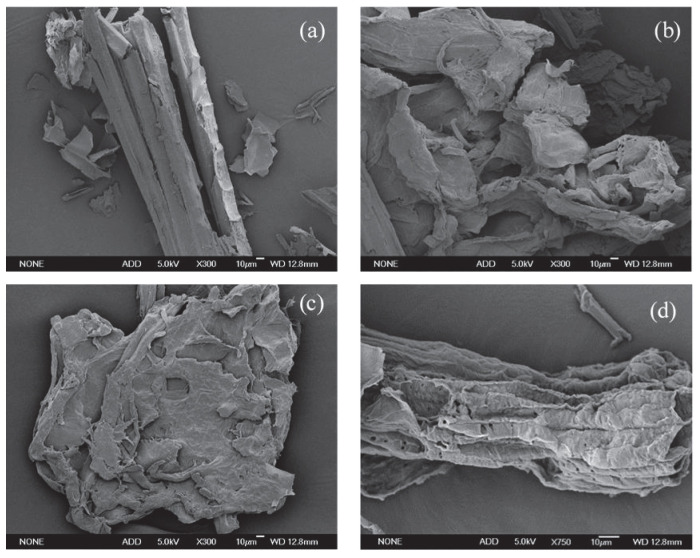
SEM images of straw after different treatments. (**a**) untreated; (**b**) NaOH combined with ozone (NaOH-ozone)-treated; (**c**) RBL at fourth cycle combined with ozone (RBL4-ozone)-treated; (**d**) XAD-16N adsorbed RBL at fourth cycle combined with ozone (ARBL4-ozone)-treated.

SEM analysis was used to provide information on the changes in surface morphological features caused by treatments. The SEM images of untreated (A), NaOH-ozone, RBL4-ozone, and ARBL4-ozone (B–D)-treated materials are presented in Figure 6.

Figure 6a shows that the surface of untreated corn straw was rough and continuous, and the dense lignocellulose structure linked by cellulose, hemicellulose and lignin was not destroyed, which greatly hindered the enzymatic hydrolysis of cellulase. Figure 6b shows the corn straw treated with fresh NaOH combined with ozone. It can be observed that the waxy coat structure and siliceous protrusions on the surface of the treated straw was mostly removed, the external structure of the straw was destroyed, and the internal structure was exposed. Lignin removal and hemicellulose separation resulted in voids and depressions of different sizes on the outer surface and the exposure of cellulose. Figure 6c presents that RBL4-ozone-treated corn straw still had curl and internal structure exposure on the surface, but the degree of straw structure damage, the surface voids and depressions were decreased. In Figure 6d, the black liquor used for corn straw treatment was alkali black liquor obtained at the third cycle after XAD-16N purification. The torn part of the straw surface was peeled off, and cellulose was exposed due to the effective removal of lignin and hemicellulose, resulting in a thinner layer of straw structure with a large number of cracks, voids and depressions.

Compare with RBL4-ozone-treated, the lignin of ARBL4-ozone-treated straw was stripped a lot and the cellulose was fully exposed, which was beneficial to the contact between cellulase and cellulose. The structure of ARBL4-ozone-treated straw was effectively destroyed, compare with untreated straw. The morphology of NaOH-ozone-treated, and ARBL4-ozone-treated straw was similar and both the treatments could efficiently open up the rigid structure of corn straw. It was indicated that XAD-16 N adsorption was effective to removal the impurities in RBL and improve the alkali utilization. The corn straw was fully swelled and split by alkali penetration and then peeled off by ozone, which caused the exposure of cellulose and improve the cellulase hydrolysis.

### 2.8. XRD of Treated Corn Straw

**Figure 7:**
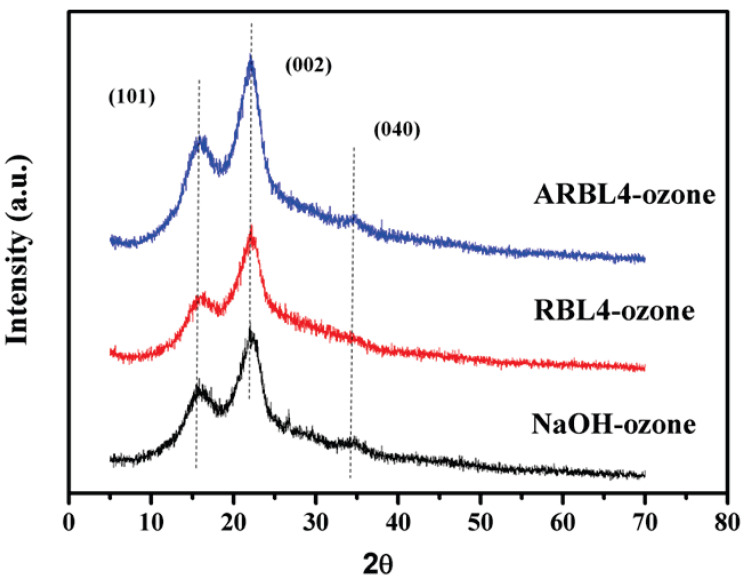
XRD of straw after different alkali black liquor combined with ozone treatments.

The cellulose of corn straw contains crystalline and amorphous regions. The cellulose crystal structure and crystallinity are important factors that affect cellulase hydrolysis [34]. XRD method is commonly used to measure the crystallinity of materials and XRD studies on corn straw before and after treated were performed. XRD analysis of different samples was presented in Figure 7. 

Three typical diffraction peaks of crystalline cellulose were observed. A peak at the diffraction angle 2θ = 20–30° is 002 crystal plane diffraction peak, a shoulder peak at 2θ = 10–20° is 101 crystal plane diffraction peak, and a peak with relatively low diffraction intensity at 2θ = 30–40° is 040 crystal plane diffraction peak. The amorphous part includes hemicellulose, lignin, and amorphous cellulose. The region around 21.5° reflected the amorphous area of cellulose. *CrI* value is influenced by the composition of biomass and the crystallization degree of cellulose [35]. The *CrIs* of untreated, NaOH-ozone-treated, RBL4-ozone-treated and ARBL4-ozone-treated straw samples were 89.31%, 47.68%, 51.85% and 50.71%, respectively. It was clear that corn straw treated with alkali black liquor before and after adsorption combined with ozone led to decrease of *CrI* compare to untreated sample, indicating that the treatment of alkali black liquor combined with ozone had destructive effects on the amorphous and crystalline regions of cellulose. The decrease of crystallinity might attribute to the removal of lignin and hemicellulose in amorphous structures, partial hydrolysis of crystalline cellulose and the increase of amorphous cellulose region, which were key factors to enhance the cellulase hydrolysis as well [36]. 

It can be found that the *CrI* of RBL4-ozone-treated corn straw was lower than that of NaOH-ozone-treated, speculating that the reduced effective alkali content in the RBL [5] resulted in the inferior destruction ability to the crystalline cellulose compared with NaOH-ozone treatment. The *CrI* of straw treated by ARBL4-ozone was between that of RBL4-ozone and NaOH-ozone treated, indicating that XAD-16N effectively adsorbed impurities such as lignin and small molecule organic matters in the alkali black liquor, which improved the alkali utilization rate. The destructive effect on crystalline cellulose was better than that of non-adsorbed combined with ozone treatment, but lower than that of NaOH-ozone treatment, which was consistent with the change trend of cellulase hydrolysis the straw treated with corresponding treatment. The increased degree of destruction of the crystalline cellulose can facilitate the contact of cellulase and cellulose, and thus enhance the efficiency of the cellulase hydrolysis.

## 3. Materials and Methods 

### 3.1. Materials

Corn straw was collected from a farm in Siping, Northeast China. The raw materials were dried at 50 °C for 48 h, crushed, screened through a 60-mess sieve and extracted with toluene and ethanol at the ratio of 2:1 for 3 h, then washed thoroughly with distilled water and dried to constant weight. The cellulose, hemicellulose, and lignin contents of the raw corn straw were measured by two-step acid hydrolysis [37] which were 39.30%, 23.40% and 27.31%, respectively. The black liquor used in this experiment was the alkali black liquor obtained at the third cycle. Its preparation can be referred to Zhou et al. [5]. The three XAD adsorbents in this study were Amberlite XAD-4, Amberlite XAD-16N, and Amberlite XAD-7HP (Rohm and Haas). Other chemicals were of analytical grade.

It was noted that the treatment of fresh sodium hydroxide (NaOH) treated with the straw was marked as the zeroth cycle, and the first cycle was carried out using the black liquor generated by the zeroth treatment, and so on. The alkali black liquor that obtained at the third cycle, was used to treat with corn straw, which called the fourth cycle (RBL4). RBL4-ozone represents the treatment of RBL4 combined with ozone. The alkali black liquor obtained at the third cycle after XAD-16N adsorption was used to treat with corn straw, which called the adsorbed fourth cycle (ARBL4). ARBL4-ozone represents the treatment of the ARBL4 combined with ozone.

### 3.2. Adsorbent Pretreatment

XAD-4 and XAD-16N resins are hydrophobic styrene-divinylbenzene adsorbent, which are suitable for adsorbing hydrophobic molecules from polar solvents. The XAD-7HP resin is an uncharged polyacrylate adsorbent which can be applicable for the adsorption of non-polar compounds from aqueous media and polar compounds from non-polar solvents. XAD-16N can be used for adsorption of hydrophobic compounds with molecular weight up to 40,000 g/mol and XAD-7HP can be used for adsorption of organic compounds with molecular weight up to 60,000 g/mol [17,28]. Physical properties of the adsorbents are listed in Table 3.

The polymeric adsorbent was soaked in anhydrous ethanol at a resin to ethanol ratio (*w/v*) of 1:3 for 24 h. The resins were washed thoroughly with deionized water, drained and stored at room temperature for future use. The used resins were washed with deionized water for three times and immersed in anhydrous ethanol which should be changed every 4 h until colorless. Then the resins were rinsed with 4% NaOH at 1:3 resin to NaOH ratio (*w/v*), subsequently washed with deionized water until the pH value was neutral. The drying and storage processes were the same as the above.

### 3.3. Batch Adsorption

#### 3.3.1. Effect of Adsorbent Time on the Extent of Removal of Lignin

In the adsorption experiments, 3 g of each adsorbent was added into 20 mL alkali black liquor. The mixtures were placed into a constant temperature bath oscillator of 150 rpm at 28 °C and equilibrated for 1 h, 1.5 h, 2 h, 2.5 h, 3 h, and 4 h, respectively. The lignin contents in the alkali black liquors were determined after the equilibration.

#### 3.3.2. Effect of Adsorbent Dose on the Extent of Removal of Lignin

Different adsorbent doses (1.0, 2.0, 3.0, 4.0, 5.0, and 6.0 g) of the three resins were added into 20 mL alkali black liquor. The mixtures were placed into a constant temperature bath oscillator of 150 rpm at 28 °C. The equilibrated time of the three adsorbents was referred to the best conditions obtained in Section 3.3.1. The lignin contents in the alkali black liquors were determined after the equilibration.

### 3.4. Adsorption kinetic Analysis

#### 3.4.1. Kinetics Model

Adsorption kinetics can be used to determine the adsorption rate. The related equations were presented as follows.

(1) $$\mathrm{The}\;\mathrm{pseudo}-\mathrm{first}-\mathrm{order}\;\mathrm{kinetics}\;\mathrm{model}:\;q_{t}=q_{e}\left(1-e^{-k_{1}t}\right)$$

(2) $$\mathrm{The}\;\mathrm{pseudo}-\sec\mathrm{ond}-\mathrm{order}\;\mathrm{kinetics}\;\mathrm{model}:\;\frac{t}{q_{t}}=\frac{1}{k_{2}q_{e}^{2}}+\frac{1}{q_{e}}t$$

where *q_t_* is the adsorption capacity at time t (mg/g); *q_e_* is the adsorption capacity of the adsorbent (mg/g); *k*_1_ and *k*_2_ are the rate constant of pseudo-first-order and pseudo-second-order adsorption, respectively.

#### 3.4.2. Intraparticle Diffusion Model

The Weber&Morris diffusion model is used commonly to reveal the mechanism of adsorption, which can be expressed as:

(3) $$q_{t}=k_{\mathrm{id}}t^{1/2}+C$$

where k_id_ is the rate constant (mg/g·min^1/2^) and C is the intercept (mg/g).

### 3.5. Adsorption Isotherms Analysis

The adsorption characteristics can be revealed by different isotherm models. Langmuir and Freundlich isotherms are two commonly used isotherm models. 

The linear of Langmuir equation was shown as follow:

(4) $$\frac{c_{e}}{q_{e}}=\frac{1}{q_{m}K_{L}}+\frac{1}{q_{m}}c_{e}$$

where *C_e_* is the final concentration of adsorbate in the bulk solution (mg/mL); *q_m_* is the maximum saturated adsorption capacity (mg/g); *K_L_* is Langmuir constant (L/g).

The Freundlich model can be expressed as:

(5) $$\;lg\;q_{e}=lg\;K_{F}+\frac{1}{n}\;lg\;c_{e}$$

where *K_F_* and *n* are both the Freundlich constants. The equation 1/*n* < 1 represents a favorable adsorption, 1/*n* > 1 represents a cooperative adsorption.

### 3.6. Determination of Lignin Content in Alkali Black Liquor

20 mL of alkali black liquors before and after the adsorption with different resins were collected and hydrolyzed with 560 mL of sulfuric acid (3%) and autoclaved for 1 h at 121°C. The autoclaved samples were filtered and the residue was washed by hot distilled water to neutral, then dried in the oven at 105 °C to constant to obtain acid insoluble lignin. The acid-soluble lignin concentration in the filtrate was determined by measuring absorbance at 205 nm.

(6) $$B=\frac{A\times D}{110}$$

where *A* requests the absorbance at 205 nm of lignin solution; *B* requests the concentration of lignin (g/L); *D* requests filtrate dilution factor; 110 requests absorbance coefficient, L/(g × cm).

The lignin removal rate was calculated using the following equation:

(7) $$\mathrm{lignin}\;\mathrm{removal}\;\mathrm{rate}\;\left(\%\right)=\frac{C_{i}-C_{e}}{C_{i}}\times 100$$

where *C_i_* is the initial concentration of the content in the alkali black liquor (mg/mL).

The adsorption capacity of adsorbent was calculated as follows:

(8) $$q_{e}=\frac{\left(C_{i}-C_{e}\right)-V}{m}$$

where *V* is the volume of the alkali black liquor (mL); m is the weight of adsorbent (g).

### 3.7. Purified Alkali Black Liquor Combined with Ozone Pretreatment of Corn Straw

Purified alkali black liquor was obtained after XAD-16N adsorption and used to treat fresh corn straw.

The purified alkali black liquor after was supplemented to the initial volume and concentration for new treatment of fresh corn straw. The alkali concentration was determined with acid-base titration. 2 g of dried corn straw were weighed accurately and reacted with 30 mL of purified alkali black liquor in a rotating water bath at 80 °C for 2 h and stirred at 150 rpm/min. After treatment, the mixture was cooled to room temperature and then filtered to separate filtrate and solid residue. The residues were washed thoroughly by distilled water to neutrality and dried at 55 °C for further analysis.

2 g of corn straws after the above treatment were weighed and soaked completely for 24 h by adding 30 mL deionized water. The mixture was added NaOH to adjust pH to 9, and then reacted with ozone (78 mg/L) for 25 min. After reaction, the treated corn straw was collected and washed completely, then dried at 55 °C to constant weight.

### 3.8. Enzymatic Hydrolysis

0.2 g of the treated corn straw mixed with 60 mL of acetate-sodium acetate buffer (0.1 mol/L pH 4.8), 30 μL cycloheximide, 40 μL tetracycline hydrochloric acid and 40 μL xylanase (45.8 U/mL) were added into an erlenmeyer flask of 100 mL. Then the mixture was incubated in a rotating water bath at 70 °C for 24 h and stirred at 120 rpm/min. After that, 40 µL cellulase (77.8 U/mL) and 30 µL β-glucosidase (690.4 U/mL) were added into the mixture that reacted at 50 °C in a shaker bath at 120 rpm/min for 72h. The enzymatic hydrolysate was collected to determine the glucose content. All assays were performed triplicate. The conversion of cellulose to glucose was calculated as follows:

(9) $$\mathrm{Conversion}\;\mathrm{rate}\;\mathrm{of}\;\mathrm{cellulolytic}\;\mathrm{hydrolysis}\;\left(\%\right)\;=\frac{C\times V_{c}\times 0.90}{m_{c}\times W}\times 100$$

where *C* is the concentration of glucose (mg/mL); *V_c_* is the total volume (mL); 0.90 is glucose conversion coefficient; *m_c_* represents the quality of corn straw (mg); *W* is percentage of cellulose content in treated corn straw.

### 3.9. Scanning Electron Microscopy (SEM) Analysis

The treated corn straw was dried in an oven at 50 °C for 24 h. The sample was mounted on conductive adhesive tape, coated with gold for 60 s and detected using a scanning electron microscope NeoScope JCM-5000 (Nikon5, Tokyo, Japan).

### 3.10. X-ray Diffraction (XRD) Analysis

The cellulose crystallinity of the pretreated corn straw was analyzed by XRD using copper-palladium radiation (λ = 0.154 nm). The operating voltage and current were 40 kV and 40 mA, respectively. The measurement method was adopted θ/2θ linkage scanning, the diffraction angle of 2θ ranged from 5° to 70° in step size of 0.02° and the step speed was 0.2 s/step. The crystallinity of cellulose was calculated according to the peak intensity method [38].

(10) $$CrI=\frac{I_{002}-I_{am}}{I_{002}}\times 100$$

where *CrI* represents the crystallinity index, *I*_002_ represents the maximum intensity of the 002 peak, and *I_am_* represents the minimum intensity between the 002 and the 101 peaks.

## 4. Conclusions

RBL was rich in lignin and small molecule organic matter. In order to increase the circulation times, three polymeric resins were applied for the purification of the RBL. Alkali black liquor recycled at the third time was used as the adsorbate. The results showed that XAD-16N adsorption with the highest of delignification rate, followed by XAD-4 and XAD-7HP. The optimal conditions of XAD-16N adsorption were 5 g/20 mL dosage for 2.5 h. Equilibrium experiments showed XAD-4 adsorption was found to was follow the Langmuir model, while XAD-16N and XAD-7HP adsorptions were fitted the Freundlich model. The adsorption kinetics data of XAD-16N and XAD-7HP resins was well fitting the Pseudo-second-order model. The use of XAD-16N was further investigated. The cellulase hydrolysis rate of corn straw treated with alkali black liquor after XAD-16N adsorption combined with ozone was 13.92% higher than that of non-adsorption treatment. SEM showed that there were tears, curling and voids on treated straw surface, and XRD showed the removal of lignin in amorphous region and low cellulose crystallinity. In conclusion, XAD-16 resin proved to be a promising adsorbent for RBL purification.

## Figures and Tables

**Table 1:** Kinetics parameters of each resin for the adsorption of lignin from alkali black liquor.

Type	Pseudo-First-Order		Pseudo-Second-Order	
	*k*_1_	R^2^	*k*_2_	R^2^
XAD-4	0.0135	0.8412	2.1927 × 10^−4^	0.8169
XAD-16N	0.0164	0.8898	4.5462 × 10^−4^	0.9476
XAD-7HP	0.0193	0.7502	1.3257 × 10^−3^	0.9064

**Table 2:** Isotherm model parameters of each resin for the adsorption of lignin from alkali black liquor.

Type	Langmuir Isotherm			Freundlich Isotherm		
	q_m_ (mg/g)	K_L_ (mL/mg)	R^2^	1/n	K_F_ (mL/g)	R^2^
XAD-4	23.5239	1.3044	0.9297	0.2490	14.0007	0.8014
XAD-16N	44.3066	0.8425	0.8346	0.4003	20.0014	0.8526
XAD-7HP	−8.2088	−0.1882	0.6213	2.4806	0.7257	0.7738

**Table 3:** Physical properties of the Amberlite polymeric resins used in this study.

Adsorbents	Matrix	Type	Particle Size (mm)	Specific Surface Area (m^2^/g)	Uniformity Coefficient D90/D40	Pore Size (Å)
XAD 4	PS-DVBa	Non-polar	0.49–0.69	≥750	≤2.00	100
XAD 16N	PS-DVBa	Non-polar	0.56–0.71	≥800	≤2.00	150
XAD 7HP	Polyacrylate	Non-polar	0.56–0.71	≥380	≤2.00	450
